# Neonatal Organophosphorus Pesticide Exposure Alters the Developmental Trajectory of Cell-Signaling Cascades Controlling Metabolism: Differential Effects of Diazinon and Parathion

**DOI:** 10.1289/ehp.0901237

**Published:** 2009-09-24

**Authors:** Abayomi A. Adigun, Nicola Wrench, Frederic J. Seidler, Theodore A. Slotkin

**Affiliations:** Department of Pharmacology and Cancer Biology, Duke University Medical Center, Durham, North Carolina, USA

**Keywords:** adenylyl cyclase, β-adrenergic receptor, cyclic AMP, diazinon, heart development, liver development, muscarinic receptor, organophosphorus insecticides, parathion

## Abstract

**Background:**

Organophosphorus pesticides (OPs) are developmental neurotoxicants but also produce lasting effects on metabolism.

**Objectives/methods:**

We administered diazinon (DZN) or parathion (PRT) to rats on postnatal days 1–4 at doses straddling the threshold for systemic signs of exposure and assessed the effects on hepatic and cardiac cell signaling mediated through the adenylyl cyclase (AC) cascade.

**Results:**

In the liver, DZN elicited global sensitization, characterized by parallel up-regulation of AC activity itself and of the responses to stimulants acting at β-adrenergic receptors, glucagon receptors, or G-proteins. The effects intensified over the course from adolescence to adulthood. In contrast, PRT elicited up-regulation in adolescence that waned by adulthood. Superimposed on these general patterns were effects on glucagon receptor coupling to AC and on responses mediated through the G_i_ inhibitory protein. The effects on the liver were more substantial than those in the heart, which displayed only transient effects of DZN on AC function in adolescence and no significant effects of PRT. Furthermore, the hepatic effects were greater in magnitude than those in a brain region (cerebellum) that shares similar AC cascade elements.

**Conclusions:**

These findings indicate that OPs alter the trajectory of hepatic cell signaling in a manner consistent with the observed emergence of prediabetes-like metabolic dysfunction. Notably, the various OPs differ in their net impact on peripheral AC signaling, making it unlikely that the effects on signaling reflect their shared property as cholinesterase inhibitors.

It is increasingly clear that adverse events during fetal development contribute to subsequent morbidity in adulthood. Although Barker (2003) originally showed that intrauterine stress sufficient to cause growth retardation contributed substantial risk to subsequent development of cardiovascular disease and diabetes, more recent results suggest that environmental contaminants, drugs, and chemicals can produce similar adverse outcomes, without growth restriction. We and others have been exploring how exposures to common pesticides might provide a route to later-emerging disorders, in particular focusing on organophosphorus pesticides (OPs), which represent 50% of worldwide insecticide use (Casida and Quistad 2004). Because pregnant women are typically exposed to OPs under circumstances that do not elicit outward signs of intoxication, and in light of recent findings suggesting that such exposures can produce long-term neurobehavioral deficits in children (Eskenazi et al. 2008; Rauh et al. 2006; Rosas and Eskenazi 2008), the mechanisms and consequences of apparently subtoxic OP exposures remain major environmental concerns.

Although the systemic toxicity and signs of OP intoxication reflect their shared ability to inhibit cholinesterase (Mileson et al. 1998), recent data indicate that lower-level OP exposures disrupt cell-signaling cascades that control cell differentiation and other critical regulatory functions (Slotkin 2005). Chief among these is the pathway that generates cyclic AMP (cAMP). This critical second messenger is controlled through neurotransmitter and hormonal receptors that link through G-proteins to regulate adenylyl cyclase (AC), the enzyme that synthesizes cAMP from ATP. Chlorpyrifos (CPF), the most studied OP, targets the AC cascade at multiple points, including the number and activity of G-protein–coupled receptors, the concentration and function of the G-proteins, and the expression and catalytic activity of AC itself (Curtin et al. 2006; Huff et al. 2001; Meyer et al. 2004; Song et al. 1997). However, AC signaling is critically important in the development and function of cells and organs besides the central nervous system and is notably involved in the control of metabolic and cardiovascular processes. Indeed, we showed that neonatal CPF exposure dysregulates hepatic and cardiac AC (Meyer et al. 2004), with global sensitization of hepatic AC, augmenting the responses to multiple neurohumoral inputs and leading ultimately to metabolic abnormalities resembling those in prediabetes (Slotkin et al. 2005).

Because OP effects on cell signaling do not necessarily involve their shared property as cholinesterase inhibitors, there is no reason to presuppose that all OPs will act in the same way. Indeed, neonatal exposure to parathion (PRT) also elicits metabolic changes related to prediabetes (Lassiter et al. 2008), but the specific defects differ from those seen with comparable exposure to CPF (Slotkin et al. 2005); further, patterns of weight gain and loss after neonatal exposure to diazinon (DZN) also diverge from those evoked by CPF or PRT (Lassiter and Brimijoin 2008; Lassiter et al. 2008; Roegge et al. 2008). Accordingly, in the present study, we examined whether the diverse outcomes of early-life exposure to different OPs are associated with differences in their long-term effects on AC signaling. We evaluated function at each step in the cascade in liver and heart [see schematic in Supplemental Material, available online (doi:10.1289/ehp.0901237.S1 via http://dx.doi.org/)] after neonatal exposure to DZN and PRT over periods ranging from early adolescence [postnatal day 30 (PND30)] through young adulthood (PND60) and late adulthood (PND100). In addition to assessing the effects on basal AC activity, we evaluated the response to β-adrenergic receptors (βARs) and glucagon receptors, both of which stimulate AC via activation of the stimulatory G-protein (G_s_). We also determined the effect of fluoride, which evokes maximal activation of both G_s_ and the corresponding inhibitory protein (G_i_). We then measured the maximal activation of AC itself by forskolin, which acts directly on the enzyme by binding to the catalytic core (Hurley 1999). Further, we measured ligand binding for βARs and for the inhibitory m_2_-muscarinic acetylcholine receptors (m_2_AChRs). We compared the effects of each OP on the trajectory of development of hepatic and cardiac AC signaling with those in the cerebellum, a brain region that shares the same high degree of AC response to βAR stimulation (Slotkin et al. 1998).

## Materials and Methods

### Animal treatments

All experiments were carried out humanely and with regard for alleviation of suffering, with protocols approved by the Institutional Animal Care and Use Committee and in accordance with all federal and state guidelines. Timed-pregnant Sprague-Dawley rats (Charles River, Raleigh, NC) were housed in breeding cages, with a 12/12-hr light/dark cycle and free access to food and water. On the day of birth, all pups were randomized and redistributed to the dams with a litter size of 10 to maintain a standard nutritional status. Randomization within their respective treatment groups was repeated at intervals of several days. In addition, dams were rotated among litters to distribute any maternal caretaking differences randomly across litters and treatment groups. Offspring were weaned on PND21. Because of their poor water solubility, DZN and PRT (Chem Service, West Chester, PA) were dissolved in dimethyl sulfoxide (DMSO) to provide consistent absorption (Whitney et al. 1995) and were injected subcutaneously in a volume of 1 mL/kg once daily on PNDs 1–4. Control animals received equivalent injections of DMSO vehicle, which does not itself produce developmental toxicity (Song et al. 1997; Whitney et al. 1995). At PND30, PND60, and PND100, one male and one female from each final litter assignment were weighed and decapitated, and the heart, one liver lobe, and the cerebellum were dissected, blotted, frozen in liquid nitrogen, and maintained at −45°C. Other tissues and brain regions were reserved for future studies. The animals used here were part of a much larger cohort used in our previous studies with DZN and PRT (Lassiter et al. 2008, 2010; Roegge et al. 2008; Timofeeva et al. 2008a, 2008b).

The dosing paradigms were chosen to achieve a toxicodynamic match between DZN and PRT (Slotkin et al. 2006a, 2006b) and, similarly, to match earlier studies using CPF (Song et al. 1997). For each OP, we chose doses that are toxicodynamically equivalent, as assessed through measurements of brain cholinesterase inhibition: 0.5 and 2 mg/kg/day for DZN, and 0.1 and 0.2 mg/kg/day for PRT.

### Assays

All procedures and materials used in this study were previously presented in detail (Meyer et al. 2004) and are described briefly here. Cell membrane preparations were assayed for AC activity in a two-step procedure, with an enzymatic step generating cAMP and subsequent analysis using commercial kits. In addition to assessing basal AC activity, we evaluated responses to 100 μM isoproterenol, 3 μM glucagon, 10 mM sodium fluoride, and 100 μM forskolin, concentrations shown in our earlier work to be maximally effective for AC stimulation. βAR binding was assayed in the same membrane preparation, using 67 pM [^125^I]iodopindolol as the radioligand and 100 μM isoproterenol to displace specific binding. For m_2_AChR binding, we used 1 nM [^3^H]AFDX384, with or without 1 μM atropine. For the ligand binding determinations, technical limitations were imposed by the large number of membrane preparations that had to be examined. The overall strategy was to determine binding at a single subsaturating ligand concentration to enable the detection of changes that originate in alterations of either *K_d_* (dissociation constant) or *B*_max_ (maximum binding).

### Data analysis

Data are presented as means ± SEs obtained from six animals in each treatment group for each sex and age point. To establish treatment differences, we first conducted a global analysis of variance (ANOVA; data log-transformed because of heterogeneous variance across ages, tissues, and AC stimulant) for all variables: the *in vivo* treatment groups (control vs. low and high OP doses), which OP was given (DZN vs. PRT), age, sex, tissue, and the stimulant condition (repeated measure). As justified by significant interactions of treatment with the other variables, data were then subdivided into lower-order ANOVAs, followed, where appropriate, by Fisher’s protected least significant difference test to identify individual values for which the OP groups differed from the control. Significance for main treatment effects was assumed at *p* < 0.05. However, for interactions at *p* < 0.1, we also examined whether lower-order main effects were detectable after subdivision of the interactive variables (Snedecor and Cochran 1967). The criterion for interaction terms was used not to assign significance but rather to identify interactive variables requiring subdivision for lower-order tests. Where treatment effects were not interactive with other variables, we report only the main treatment effects without performing lower-order analyses.

To enable ready visualization of treatment effects across different tissues, ages, and stimulants, the results are given as the percent change from control values, but statistical procedures were always conducted on the original data. Control values are shown in the Supplemental Material, Table 1, (doi:10.1289/ehp.0901237.S1)], which also shows the highly significant increase in AC evoked by each of the stimulants.

## Results

Multivariate ANOVA incorporating all age points and both sexes showed that neonatal OP exposure elicited small but statistically significant effects on body weight (main treatment effect, *p* < 0.004) and heart weight (*p* < 0.0001); liver weights could not be compared because we dissected only a single lobe. The effects on body and heart weight depended on which OP was given, as evidenced by significant interactions of treatment × (DZN vs. PRT): *p* < 0.02 for body weight, *p* < 0.005 for heart weight. DZN produced a significant overall reduction in body weight at either 0.5 or 2 mg/kg, amounting to net deficits of 5% (*p* < 0.003) and 6% (*p* < 0.0006), respectively (data not shown), consistent with earlier findings (Roegge et al. 2008); for heart weight, the reductions were slightly larger, 8–9% (*p* < 0.0001 for either dose). In contrast, for PRT we found no statistically significant effects on body or heart weights in the animals used for this study (data not shown); however, these animals were part of a much larger cohort that was used for additional studies (Lassiter et al. 2008; Slotkin et al. 2009), and in the overall group, PRT caused a small (2–3%) but significant elevation in body weight at the low dose in males and reductions of about 4% at either dose in females.

Global ANOVA for AC measurements indicated a significant main effect of OP treatment reflecting overall increases in activity (*p* < 0.0001; OP treated > control) that differed between liver and heart (treatment × tissue, *p* < 0.0001) and between DZN and PRT [treatment × (DZN vs. PRT), *p* < 0.04], as well as displaying more complex interactions: *p* < 0.004 for treatment × (DZN vs. PRT) × age; *p* < 0.02 for treatment × (DZN vs. PRT) × age × tissue; *p* < 0.0001 for treatment × AC stimulant; and *p* < 0.0001 for treatment × tissue × AC stimulant. Accordingly, for presentation we separated the data into the individual tissues (liver, heart, cerebellum) and treatments (DZN, PRT) and then performed lower-order tests to reexamine the results for treatment effects and interactions.

### Liver AC

In the liver, neonatal OP exposure elicited a significant main treatment effect (*p* < 0.0001) that differed between the two OPs and among ages [treatment × (DZN vs. PRT), *p* < 0.08; treatment × (DZN vs. PRT) × age, *p* < 0.0001] and also showed selectivity among the various AC stimulants (treatment × AC stimulant, *p* < 0.0001). Accordingly, we separated the results for DZN and PRT for each age point and then reexamined the treatment effects.

**Figure 1:**
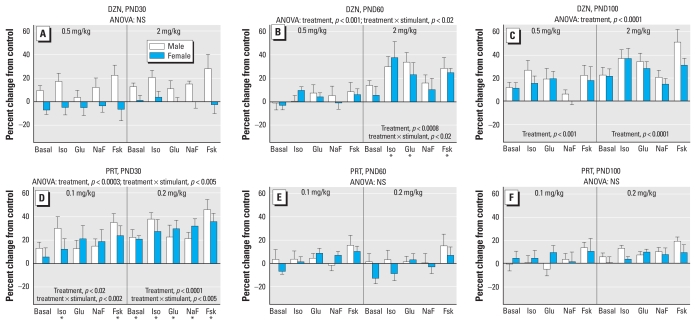
Effects of neonatal exposure to DZN (*A*–*C*) or PRT (*D*–*F*) on liver AC activity on PND30 (*A, D*), PND60 (*B, E*), and PND100 (*C, F*). Abbreviations: Fsk, forskolin; Glu, glucagon; Iso, isoproterenol; NaF, sodium fluoride; NS, not significant. ANOVA incorporating the factors of treatment, sex, and AC stimulant appears at the top of each panel, and lower-order tests are shown within the panels. Where there was a significant treatment × stimulant interaction, asterisks denote specific responses that differ from the control animals (males and females combined); separate tests for males and females were not carried out because of the absence of treatment × sex interactions.

DZN exposure elicited a significant main treatment effect (*p* < 0.0001), reflecting an overall gain of function, with dependence on age (treatment × age, *p* < 0.08) and AC stimulant (treatment × stimulant, *p* < 0.005). On PND30 we observed a trend toward overall increases in AC activity in males that did not by itself achieve statistical significance (Figure 1A); however, we saw the same pattern in the heart, and the overall effect across both tissues was significant. By young adulthood (PND60), the main effect of DZN treatment became significant for both sexes, reflecting a robust enhancement of activity in the animals exposed to 2 mg/kg (Figure 1B). Superimposed on this general increase, there were selectively greater effects on responses to stimulants acting through G_s_-coupled receptors (isoproterenol, glucagon) than on the response to fluoride, which stimulates both G_s_ and G_i_; the response to forskolin was also increased to a greater extent than that to fluoride. By PND100 (Figure 1C), both the low and high doses of DZN elicited significant increases in AC. Like the PND60 values, the fluoride response was affected to a lesser extent than that to the receptor stimulants or forskolin (*p* < 0.002 for treatment × stimulant across the two age points).

For hepatic effects of PRT exposure, the global ANOVA indicated a significant main treatment effect (*p* < 0.0009) that depended on age (treatment × age, *p* < 0.003), AC stimulant (treatment × stimulant, *p* < 0.0001), and sex (treatment × stimulant × sex, *p* < 0.06). Most notably, the temporal pattern of effects was completely distinct from that of DZN. On PND30 (Figure 1D), neonatal PRT exposure elicited significant up-regulation of AC activity at both 0.1 mg/kg and 0.2 mg/kg. Superimposed on the overall increase, the lower dose elicited selectively greater effects on the responses to isoproterenol and forskolin than on those to glucagon or fluoride; we saw the same pattern at the higher dose, although the greater overall effect increased the response sufficiently to achieve statistical significance for all the individual measures. On PND60, the point at which DZN elicited even greater increases in hepatic AC activity, PRT instead showed a loss of effect (Figure 1E), and the same was true in full adulthood, by PND100 (Figure 1F). To make certain that later- emerging changes were not occurring with PRT exposure, we performed an additional set of determinations at 5 months of age and again found no persistent overall effects on AC (data not shown).

### Heart AC

**Figure 2:**
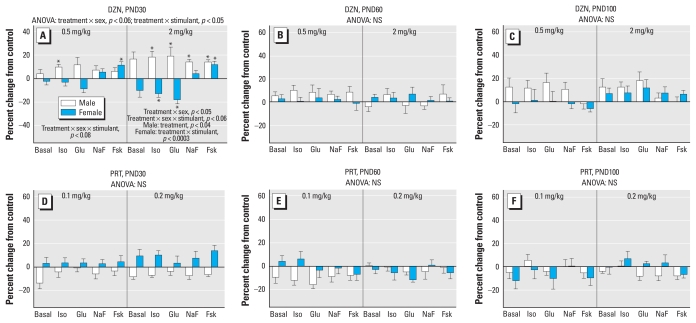
Effects of neonatal exposure to DZN (*A*–*C*) or PRT (*D*–*F*) on heart AC activity on PND30 (*A*, *D*), PND60 (*B*, *E*), and PND100 (*C*, *F*). Abbreviations: Fsk, forskolin; Glu, glucagon; Iso, isoproterenol; NaF, sodium fluoride; NS, not significant. ANOVA incorporating the factors of treatment, sex, and AC stimulant appears at the top of each panel, and lower-order tests are shown in (*A*). Where there was a significant treatment × stimulant interaction, asterisks denote specific responses that differ from the control animals.

In the heart, the effects of neonatal toxicant exposure were less statistically robust than for the liver, but we nevertheless found differences between the two OPs [treatment × (DZN vs. PRT), *p* < 0.07], as well as treatment interactions with age and AC stimulant (treatment × age × AC stimulant, *p* < 0.04). Again, values were separated into the individual treatments and ages for comparisons of lower-order treatment effects. On PND30 (Figure 2A), DZN treatment enhanced AC activity in a sex- and stimulant-selective manner. At the lower dose, we observed small overall effects that achieved statistical significance for 2 of the 12 parameters. More robust effects were evident at the higher dose, reflecting a net overall increase in males; in females, the responses to isoproterenol and glucagon were decreased by DZN exposure, whereas the response to fluoride was unchanged and that to forskolin was increased. The augmented responses seen in males were similar in direction and magnitude to those noted for the liver at the same age (Figure 1A); ANOVA incorporating both tissues confirmed a main effect of DZN treatment (*p* < 0.008) without tissue selectivity (no treatment × tissue interaction). By PND60, we no longer detected significant effects of DZN on heart AC activity (Figure 2B), although a slight enhancement reappeared by PND100 that did not achieve statistical significance (Figure 2C).

In contrast to the effect of DZN on heart AC, we did not detect significant effects of PRT on the same parameters (Figure 2D–F). Again, we performed a follow-up study at 5 months of age to determine if alterations might emerge even later on but found no significant treatment effects (data not shown).

### Receptors

**Figure 3:**
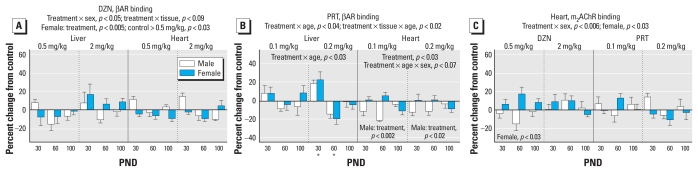
Effects of neonatal DZN or PRT exposure on liver and heart receptors. (*A*) βAR binding in DZN-exposed animals. (*B*) βAR binding in PRT-exposed animals. (*C*) Cardiac m_2_AChR binding in animals exposed to DZN or PRT. ANOVA incorporating the factors of treatment, sex, age, and tissue appears at the top of each panel, and lower-order tests are shown within the panels. Asterisks in (*B*), where there was a treatment × age interaction, show ages for which the PRT group differs from the control.

For βARs, global ANOVA identified interactions of treatment × age (*p* < 0.004), treatment × sex (*p* < 0.05), treatment × tissue (*p* < 0.09), treatment × (DZN vs. PRT) × sex (*p* < 0.01), treatment × (DZN vs. PRT) × tissue (*p* < 0.1), and treatment × (DZN vs. PRT) × tissue × age (*p* < 0.08). Again, we separated the values for the DZN and PRT treatments and examined lower-order main treatment effects and interactions of treatment with other variables. For DZN (Figure 3A), we found treatment interactions with sex and tissue. Separation of the values by tissue did not reveal any individually significant treatment effects. However, separation by sex confirmed an overall significant reduction in βAR binding in females exposed to the lower dose of DZN; this effect had a magnitude of < 10%, far smaller than the 30–40% changes seen for the AC response to βAR stimulation, and was in the opposite direction (decrease for βARs, increase for AC response). Neonatal PRT treatment also evoked significant changes in βAR binding that depended on age and tissue (Figure 3B). Separate analyses for each tissue indicated an age-dependent effect in the liver, reflecting an increase on PND30 and a decrease on PND60, restricted to the high-dose group. In the heart, βAR binding was decreased selectively in males.

We evaluated m_2_AChR binding only in the heart because these receptors are sparse in the liver and cerebellum. The global ANOVA identified a treatment × sex interaction, and subdivision of the results showed a significant treatment effect in females. Nevertheless, the net effect was quite small, and the only determinations that achieved statistical significance were those for 0.5 mg/kg DZN (Figure 3C).

### Cerebellum

**Figure 4:**
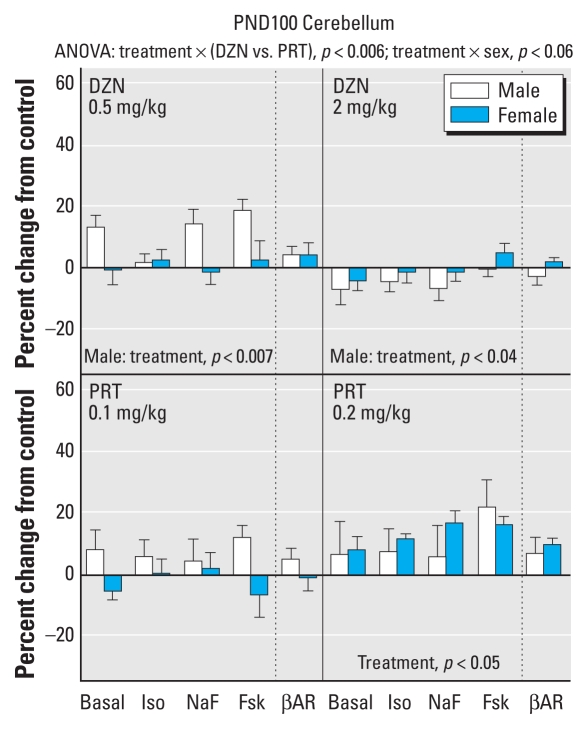
Effects of neonatal exposure to DZN (top) or PRT (bottom) on the cerebellum, evaluated at PND100. Abbreviations: Fsk, forskolin; Iso, isoproterenol; NaF, sodium fluoride. Multivariate ANOVA appears at the top of the panel, and lower-order tests are shown within the panels.

Neonatal exposure to the lower dose of DZN elicited significant increases in cerebellum AC activity that were selective for males, but the higher dose instead produced small but significant decrements (Figure 4); the magnitude of the effects was substantially smaller than that seen in the liver at the same age (PND100). The low dose of PRT did not elicit significant alterations in cerebellar AC parameters, but the high dose produced an overall increase without sex selectivity. None of the treatments had a significant effect on cerebellar βAR binding.

## Discussion

Results of this study reinforce the concept that the developmental effects of OPs extend beyond the nervous system. Neonatal exposures to DZN and PRT altered the developmental trajectory of AC-mediated cell signaling in peripheral tissues, and to a greater extent in the liver than in the cerebellum. Although, in general, OP exposure elicited a net AC gain-of-function, the effects differed among the various tissues, effectively ruling out a global effect on expression of receptors, G-proteins, or AC and instead pointing to selectively greater effects on specific aspects of hepatic function. This conclusion was further reinforced by the fact that DZN and PRT exposures evoked different trajectories despite doses of each OP chosen to be toxicodynamically equivalent (Slotkin et al. 2006b): The effects of DZN intensified with age, whereas those of PRT waned.

In our previous work with CPF, we found persistent, global increases in all measures of hepatic AC signaling, an effect restricted to males (Meyer et al. 2004); this connotes global sensitization of the pathway, wherein up-regulation of AC activity itself (enhanced forskolin response) produces an augmented response to stimulants acting on G_s_-coupled receptors (isoproterenol, glucagon), as well as to those directly activating G-proteins (fluoride). Equally important, the CPF effects displayed a critical period of sensitivity, indicating that these are specifically developmental actions (Meyer et al. 2004). As found in the present study, neonatal DZN exposure produced a similar global effect that appeared in adolescence and young adulthood at the higher dose but that also became significant at the lower dose by full adulthood; notably, although the effect emerged first in males, it eventually encompassed both sexes, thus differing in outcome from the sex-specific effects seen for CPF (Meyer et al. 2004). We also found alterations in specific elements of the signaling cascade superimposed on global sensitization. If the up-regulation of AC itself were the only effect of DZN exposure, then all pathway stimulants should show the same degree of enhancement. Instead, at both PND60 and PND100, the response to fluoride was augmented to a significantly smaller extent than that for forskolin or either of the receptor stimulants. Fluoride differs from isoproterenol and glucagon in that it also activates G_i_, and consequently, our findings point to an increase in expression and/or function of G_i_ after neonatal DZN exposure; again, this differs from the effects of CPF, which produces uniform enhancement of responses as expected from activation of AC itself (Meyer et al. 2004). Effects on G_i_ will also produce global alterations in AC signaling because they affect the response to any receptor acting through G_i_. Thus, all the effects we noted involved the signaling proteins downstream from the receptors, either AC itself or the G-proteins, rather than reflecting effects on the expression of the neurotransmitter receptors or their specific coupling to the control of AC. Indeed, none of the small effects seen for receptor binding could account for the robust augmentation of AC responses.

In contrast to DZN, neonatal PRT exposure produced a much larger initial sensitization of hepatic AC in adolescence, again involving global changes at the level of AC itself, but additional pathway effects were evident at the lower dose, reflecting smaller increments for glucagon and fluoride than for isoproterenol and forskolin. In turn, these effects imply homologous desensitization for glucagon and heterologous increases in G_i_-mediated inhibition; the effect of PRT on the glucagon response was not shared by DZN, again pointing out specific differences related to each individual OP. Further, the effects of DZN intensified over time, whereas those of PRT waned. Thus, the two OPs differ completely in their effects on the developmental trajectory of hepatic AC signaling, with large effects in adolescence for PRT but not DZN, supplanted by the opposite pattern in adulthood. The fact that AC signaling in the liver was affected far more than in either the heart or cerebellum further demonstrates selective effects of early-life OP exposure. Finally, for the heart (but not the cerebellum), we found significant changes in receptor binding without apparent connection to the effects on AC signaling, again reinforcing the importance of sensitization downstream from the receptors as the primary site of regulatory disruption.

The clear implication is that neonatal OP exposure is likely to affect hepatic responses to a greater extent than those in the heart or in the central nervous system, and it is therefore critical to examine how the cellular changes seen here might then contribute to alterations in tissue function. In the liver, where βARs and glucagon receptors are linked through AC to enhanced gluconeogenesis and lipolysis, global sensitization of AC signaling leads to corresponding metabolic abnormalities. In our earlier work with CPF, we established the presence of hyperlipidemia, but serum glucose levels were maintained within normal limits (Slotkin et al. 2005); however, glucose homeostasis was maintained only by compensatory hypersecretion of insulin, thus producing a metabolic profile akin to prediabetes. Critical to the proposed mechanistic connections, the sex selectivity of the metabolic effects (males) exactly matched that for the sensitization of hepatic AC signaling (Meyer et al. 2004). More recently, we also found evidence of prediabetes after neonatal PRT exposure (Lassiter et al. 2008). In this case, there was no corresponding increase in serum insulin, and as a result, the animals displayed a frank prediabetic profile, characterized by hyperglycemia and impaired glucose and lipid utilization (Lassiter et al. 2008). It is thus important to note that the effects of PRT on hepatic AC signaling seen here were restricted to adolescence and, unlike those of CPF, did not persist into adulthood. Accordingly, for PRT, either the AC changes are unrelated to the metabolic disorders, or the effects in adolescence may be sufficient to reprogram metabolism so that defects emerge later, despite the subsequent normalization of signaling parameters. Future work will need to dissect the temporal emergence of prediabetes after neonatal PRT exposure in order to distinguish these two possibilities. However, the present data point toward the latter interpretation, because PRT-exposed animals show a switch from enhanced to suppressed weight gain coinciding with the time course for the disappearance of the effects on AC signaling (Lassiter et al. 2008). Although detailed metabolic studies have not been done for DZN, based on the results for hepatic AC signaling, we would expect to see greater metabolic defects than for PRT, consistent with the greater weight loss seen here and in earlier studies (Lassiter et al. 2008; Roegge et al. 2008); we further predict that, unlike CPF, DZN will target metabolic function in both males and females because the cellular effects were not sex selective. Finally, human studies suggest a connection of diabetes to long-term OP exposure (Morgan et al. 1980; Saldana et al. 2007) and a link between gain-of-function AC gene polymorphisms and diabetes susceptibility (Nordman et al. 2008). Our results thus provide a mechanistic underpinning for these population studies. Nevertheless, it is clear that actions on the hepatic AC pathway are likely to be just the tip of the iceberg for the metabolic abnormalities wrought by early-life OP exposure. In our earlier work, we showed how additional stresses imposed by elevated dietary fat intake can reveal defects that likely originate in adipocyte dysfunction rather than in hepatic metabolism (Lassiter et al. 2008, 2010), and it would be worthwhile to pursue these additional contributions to prediabetes.

There are similar implications for the significant, albeit lesser, effects of DZN and PRT exposure on AC signaling in the heart and cerebellum. Transgenic animals that produce AC hyperstimulation through overexpression of βARs or G_s_ show development of cardiomyopathies and abnormal heart rate regulation (Gao et al. 2003; Iwase et al. 1996). Alterations in m_2_AChR expression, which we detected for PRT, also have corresponding effects on cardiac function and the response to autonomic input (LaCroix et al. 2008). Importantly, the fact that DZN reduced heart weight significantly and to a greater extent than body weight indicates the need to pursue potential consequences for cardiac function, which have been much less studied than hepatic function. Indeed, the prediabetic changes seen after neonatal OP exposure are themselves likely to contribute to further cardiovascular morbidities. In the cerebellum, we found a nonmonotonic effect of DZN, with enhanced AC signaling at the low dose that disappeared or was reversed at the high dose; this likely represents the positive neurotrophic effect of acetylcholine produced by a small degree of cholinesterase inhibition, offsetting the direct effects of low exposure (Timofeeva et al. 2008a). In contrast, PRT produced up-regulation with a typical, monotonic dose–effect relationship. Most important, the disparate dose–effect patterns of effects on AC signaling for both DZN and PRT correspond to the differences in behavioral findings between the two agents (Timofeeva et al. 2008a, 2008b).

Our results support the view that developmental exposure to OPs targets the trajectory of AC signaling in peripheral tissues, thus extending their actions outside the nervous system, with the consequences that *a*) the effects on signaling occur with nonsymptomatic exposures, and *b*) effects differ among OPs even at toxicodynamically equivalent exposures as assessed at the level of brain cholinesterase inhibition (Slotkin et al. 2006b). Further, the liver appears to be especially sensitive to persistent disruption of AC signaling, involving sensitization of the entire pathway via induction of AC activity. In turn, this provides a likely mechanism for the metabolic consequences of neonatal OP exposure identified in earlier studies, indicative of a prediabetic state (Lassiter et al. 2010; Slotkin et al. 2005). Indeed, global sensitization is likely to have widespread consequences in general because it affects all humoral signals that operate through cAMP, rather than involving any single input. Future studies will need to address how the tissue-selective changes in cell signaling come about, that is, whether they involve a direct impact of the OPs on expression or function of the signaling proteins, whether they are downstream events secondary to effects on differentiation of hepatocytes and myocytes, or whether they originate in upstream effects on hormonal or neuronal input. In any case, our findings extend the Barker (2003) hypothesis, which originally related prenatal growth restriction to subsequent development of cardiovascular disease and diabetes, to include otherwise nonsymptomatic chemical exposures that may produce similar outcomes without the precondition of fetal/neonatal growth restriction. Our findings point out the need to explore the possibility that developmental exposure to common chemical contaminants contributes to the explosive worldwide increase in diabetes and obesity.
